# Epidemiologic and Environmental Risk Factors of Rift Valley Fever in Southern Africa from 2008 to 2011

**DOI:** 10.1089/vbz.2015.1774

**Published:** 2015-08-01

**Authors:** Margaret M. Glancey, Assaf Anyamba, Kenneth J. Linthicum

**Affiliations:** ^1^ Johns Hopkins Bloomberg School of Public Health, Baltimore, Maryland.; ^2^ NASA/Goddard Space Flight Center, Greenbelt, Maryland.; ^3^ USDA Center for Medical, Agricultural & Veterinary Entomology, Gainesville, Florida.

**Keywords:** Rift Valley fever, Southern Africa, Environmental factors, Geographic factors, Normalized difference vegetation index data

## Abstract

***Background:*** Rift Valley fever (RVF) outbreaks have been associated with periods of widespread and above-normal rainfall over several months. Knowledge on the environmental factors influencing disease transmission dynamics has provided the basis for developing models to predict RVF outbreaks in Africa. From 2008 to 2011, South Africa experienced the worst wave of RVF outbreaks in almost 40 years. We investigated rainfall-associated environmental factors in southern Africa preceding these outbreaks.

***Methods:*** RVF epizootic records obtained from the World Animal Health Information Database (WAHID), documenting livestock species affected, location, and time, were analyzed. Environmental variables including rainfall and satellite-derived normalized difference vegetation index (NDVI) data were collected and assessed in outbreak regions to understand the underlying drivers of the outbreaks.

***Results:*** The predominant domestic vertebrate species affected in 2008 and 2009 were cattle, when outbreaks were concentrated in the eastern provinces of South Africa. In 2010 and 2011, outbreaks occurred in the interior and southern provinces affecting over 16,000 sheep. The highest number of cases occurred between January and April but epidemics occurred in different regions every year, moving from the northeast of South Africa toward the southwest with each progressing year. The outbreaks showed a pattern of increased rainfall preceding epizootics ranging from 9 to 152 days; however, NDVI and rainfall were less correlated with the start of the outbreaks than has been observed in eastern Africa.

***Conclusions:*** Analyses of the multiyear RVF outbreaks of 2008 to 2011 in South Africa indicated that rainfall, NDVI, and other environmental and geographical factors, such as land use, drainage, and topography, play a role in disease emergence. Current and future investigations into these factors will be able to contribute to improving spatial accuracy of models to map risk areas, allowing adequate time for preparation and prevention before an outbreak occurs.

## Introduction

Rift Valley fever (RVF) was first identified in 1931 in Kenya's Great Rift Valley following a large epizootic among sheep (Daubney 1931). Since that time, outbreaks have been reported across Africa and the Arabian Peninsula (Davies 1975, Swanepoel 1976, Meegan and Bailey 1989, Davies et al. 1992, Arthur et al. 1993, Madani et al. 2003, Mohamed et al. 2010, Shieh et al. 2010, Hassan et al. 2011). This disease was first recognized in South Africa in 1951, an epidemic that resulted in 500,000 abortions and 100,000 deaths among sheep (Gear et al. 1951, Woods et al. 2002). Since then, it has continued to occur every 3–15 years, with the last severe outbreak before 2008 occurring in 1970 (Pienaar and Thompson 2013). The most recent multiyear epizootic starting in 2008 and ending in 2011 resulted in approximately 303 human cases, including 26 deaths and over 19,000 cases in livestock (National Health Laboratory Systems 2011).

RVF infects a variety of wild and domestic ruminants, but livestock, specifically sheep, cattle, and goats, are the most impacted (Meegan and Bailey 1989). In sheep, abortion rates can reach 100%, with young animals and foreign breeds being most at risk for severe disease and death. In adult animals, mortality ranges from 5% to 60%. Several livestock vaccines exist; however, they all have limitations and are costly, preventing widespread use (Dungu et al. 2013).

RVF impacts a community in multiple ways. First, outbreaks are associated with huge economic loss. This is due to a combination of livestock deaths and strict trade bans imposed and enforced by the World Organization for Animal Health (OIE) preventing animal export during outbreaks (Little 2009, Rich and Wanyoike 2010). In addition to this, humans can become infected, usually resulting in mild flu-like symptoms with a 1–5% risk of developing severe complications or death (Bird et al. 2009). Finally, outbreaks commonly are accompanied with flooding, which is associated with infected vector emergence (Linthicum et al. 1999). These communities often are vulnerable, with low resilience to disease and disaster, making it imperative for public health officials to recognize and prepare for these outbreaks (Dar et al. 2013).

Current prediction models exist to create RVF risk maps based on several satellite-derived measurements including sea surface temperatures, outgoing longwave radiation, normalized difference vegetation index (NDVI), and rainfall (Anyamba et al. 2009, 2010). Excess rainfall results in flooding and hatch of dormant *Aedes* virus-infected mosquito eggs in dambo habitats that then infect livestock and humans (Linthicum et al. 1984).

NDVI data have been proven to be a good surrogate for rainfall along with soil moisture, soil type, and energy availability in the tropical semiarid regions of Sub-Saharan Africa (Nicholson et al. 1990, Tucker and Nicholson 1999). Thus, these data are used as an indicator of breeding and upsurge patterns of some insect pests and vectors of disease, including locusts and mosquitoes (Hielkema et al. 1986, Linthicum et al. 1990). In this study, we sought to apply these parameters to southern Africa's varied subtropical climate, ranging from dry desert-like conditions in the west (NDVI values of 0.1–0.2 and annual rainfall between 200 and 300 mm) to wetter and highly vegetative regions in the east (NDVI values of 0.6 and annual rainfall as high as 600–800 mm) to observe correlations with the RVF outbreaks of 2008–2011.

## Materials and Methods

### Livestock data

RVF animal case data were obtained from the OIE's World Animal Health Information Database (WAHID) by searching monthly reports from mid-2007 through 2013 (WAHID 2008, 2009a, 2009b, 2010a, 2010b, 2010c, 2011a, 2011b, 2012). Each report is location and species specific. Data from outbreaks in South Africa, Namibia, Botswana, and Swaziland were included. Epidemic years were synchronized to the seasonal rainfall cycle preceding RVF outbreaks so that 2008 contained all outbreaks between October, 2007, and September, 2008; 2009 contained all outbreaks between October, 2008 and September, 2009, and so on. Animals were tested and cases were identified using methods described by the OIE Manual (World Organization for Animal Health 2011).

### Statistics, epidemiological curves, and mapping

Average case fatality rate and risk were calculated by year. Case fatality was defined as the number of deaths divided by number of cases for each species-specific report, and risk was defined as the number of cases divided by the total number of susceptible animals for each report. Average risk is the average percent of each species infected at each location. Incidence rates were calculated by province using animal density data from the Global Livestock Production and Health Atlas (GLIPHA) (Food and Agriculture Organization of the United Nations 2013).

Epidemic curves were created by summing the total number of cases by week for each epidemic year beginning with week 40, correlating to October 1^st^, and ending with week 39, correlating to the last week of September the following year. All data management, summary statistics, and epidemic curves were done using Stata 12.0 (StataCorp 2011). Epidemic curves were created and defined using methodology described by the Centers for Disease Control and Prevention (2014).

Case locations were mapped using ArcGIS 10.0 (Environmental Systems Research Institute 2011). Data were imported, then transformed using the given latitude and longitude coordinates and projected to a geographic coordinate system using the World Geodetic System (WGS) 1984 datum. Base maps were provided by the Environmental Systems Research Institute (Esri) for the case location map and the Database of Global Administrative Boundaries (GADM) via DIVA-GIS–provided administrative boundaries for the risk maps.

### Rainfall and NDVI data

**FIG. 1.**
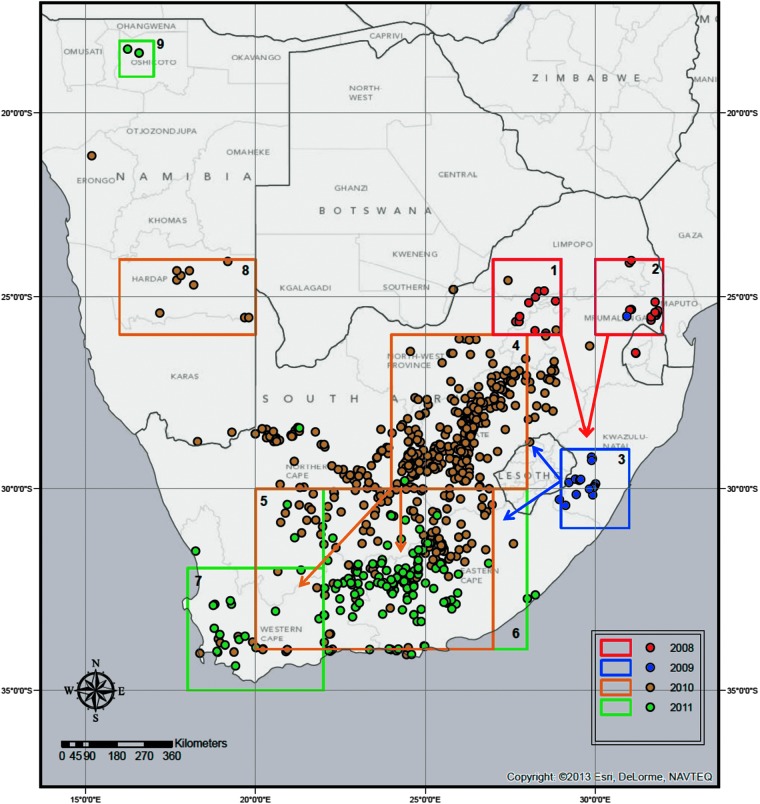
Rift Valley fever (RVF) case locations for 2008–2011 epidemic years and outlined regions with high density of cases used for the rainfall and normalized difference vegetation index (NDVI) analysis.

Rainfall and NDVI data were extracted and analyzed for nine predefined regions, (Fig. 1), corresponding to areas with a high density of RVF cases. Rainfall data were obtained from the National Oceanic and Atmospheric Administration's (NOAA's) Climate Prediction Center (CPC) Africa Rainfall Climatology (ARC) archives. These data are derived from geostationary infrared (IR) measurements centered over Africa created by the European Organization for the Exploitation of Meteorological Satellites (EUMETSAT) combined with quality controlled Global Telecommunication System (GTS) 24-h rainfall gauge observations across Africa (Novella and Thiaw 2013). Daily and long-term mean, minimum, and maximum rainfall and anomaly values were extracted for each region from 2007 to 2011.

NDVI data was derived from measurements made by the National Aeronautic and Space Administration's (NASA's) Earth Observing System Moderate Resolution Imaging Spectroradiometer (MODIS) instrument aboard the Terra (EOS AM-1) spacecraft (Townshend and Justice 2002). For this study, we used MOD13C2 NDVI data aggregated from 16 day 250 m MODIS NDVI to global monthly 0.05 degree (5600 m) NDVI data (US Geological Survey, no date). Monthly and long-term mean, minimum, and maximum NDVI and anomaly values were extracted for each region from 2007 to 2011.

Cumulative daily rainfall and monthly NDVI means were calculated by summing up all values between October 1^st^ and each succeeding time point for the epidemic year corresponding with each region. All analyses and graphs were computed and plotted using Stata 12.0 (StataCorp 2011).

## Results

There were 759 reports of RVF in southern Africa between January, 2008, and December, 2011. A total of 716 reports were from sheep, goats, or cattle, accounting for 96.8% of cases (18,767 cases out of 19,390 total cases) reported from 2008 to 2011. A total of 608 cases occurred in buffalo, camels, mixed livestock populations, or other wild ruminants. Reports of abortions and sudden or acute deaths in young lambs were also recorded.

The 2008 and 2009 outbreaks were small in magnitude compared to 2010 and 2011. In 2008, cattle accounted for 81% of cases out of 496 total livestock cases. The average number of animals infected at each location was highest during 2008, about 17%, but case fatality was lower than the following years, only about 60% versus 75–80%, and incidence rates were lower during these years ranging from 2.33 to 23.89/100,000 animals in cattle to 1.98 to 91.97/100,000 in goats. In 2009, 201 out of 210 cases occurred in cattle, and the risk of infection was only 2% (Tables 1 and 2).

**Table 1.** Rift Valley Fever Outbreak Statistics by Year of Occurrence and Species

	*Cattle*	*Goats*	*Sheep*	*Other*	*Total*
2008					
Outbreak reports	23	2	1	3	29
Cases	403	67	26	13	509
Average risk^a,b^	17.2%	29.3%	8.1%		17.4%
Average case fatality^c^	61.1%	53.2%	61.5%		60.5%
2009					
Outbreak reports	17	0	1	0	18
Cases	201	0	9	0	210
Average risk^a,b^	2.0%		9.4%		2.5%
Average case fatality^c^	60.3%		0.0%		57.0%
2010					
Outbreak reports	153	21	371	27	572
Cases	782	324	13,016	337	14,459
Average risk^a,b^	9.0%	10.7%	10.0%		9.8%
Average case fatality^c^	72.4%	61.9%	74.0%		73.1%
2011					
Outbreak reports	23	12	92	13	140
Cases	63	400	3,491	258	4,212
Average risk^a,b^	11.0%	10.8%	11.2%		11.1%
Average case fatality^c^	94.7%	77.2%	85.9%		86.6%
Total outbreak reports	216	35	465	43	759
Total sum of cases	1449	791	16,542	608	19,390
Average risk	9.2%	11.3%	10.2%		10.0%
Average case fatality	72.6%	66.6%	76.2%		74.6%

^a^ A total of 663 livestock reports containing total susceptible species counts available for risk analysis.

^b^ Average risk=mean (cases/susceptibles) from reports.

^c^ Average case fatality=mean (deaths/cases) from reports.

**Table 2.** Rift Valley Fever Incidence by Province (per 100,000 Animals)

	*2008*			*2009*			*2010*			*2011*		
*Province*	*Cattle*	*Goats*	*Sheep*	*Cattle*	*Goats*	*Sheep*	*Cattle*	*Goats*	*Sheep*	*Cattle*	*Goats*	*Sheep*
Limpopo	2.33								0.02			
Gauteng	12.60	91.97					0.38		8.56			
Mpumalanga	23.89	1.98				3.38			11.77			
Kwazulu-Natal				7.22								
Northwest	4.54		1.45				0.68		24.11			
Free State							17.22	29.38	187.97			
Eastern Cape							1.16	0.99	6.50	1.58	3.81	26.67
Northern Cape							5.07	8.22	344.18	0.06		120.99
Western Cape							38.69	15.49	12.54	2.16	108.18	25.45

In 2010 there were 14,122 cases reported in livestock; 92% occurred in sheep. The average risk of infection was 10%. In the 2011 epizootic year, there were 3954 cases in livestock, 88% in sheep. The highest incidence of infection occurred during these years in the Northern Cape province in sheep, reaching 344 cases/100,000 and 121 cases/100,000 animals in 2010 and 2011, respectively (Table 2). The average risk of infection was the same among all species and similar to 2010; however, case fatality was highest during this year for all species, reaching 95% and 86% in cattle and sheep, respectively (Table 1). Camels, buffalo, waterbuck, and a variety of other wild species were infected in addition to livestock. Overall, more of these wild species were identified during 2010 and 2011 when the outbreaks were larger in magnitude.

### Timing and location of outbreaks

**FIG. 2.**
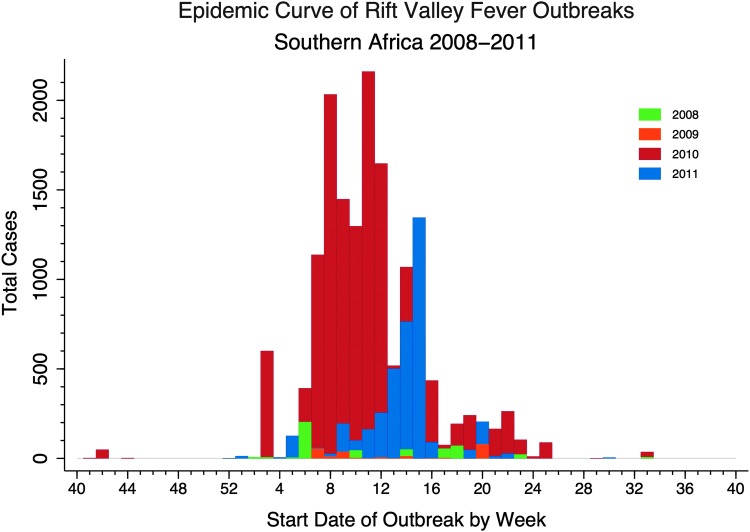
Overlapping epidemic curves for each year Rift Valley fever (RVF) occurred in southern Africa including all species. Week 40 corresponds with October 1, 2007, 2008, 2009, and 2010.

RVF cases were detected by the end of January, except in 2009, and the bulk of the cases each year occurred within a 20-week timeframe between February and June (Fig. 2). Unlike most RVF epidemics that begin and end within 1 year, this series of outbreaks continued over 4 years defined by the rainy season. In 2008 and 2009, cases occurred more sporadically and the circulating RVF virus was lineage C, but in 2010 and 2011 the outbreaks were RVF lineage H and a defined epidemic wave was evident (Grobbelaar et al. 2011).

**FIG. 3.**
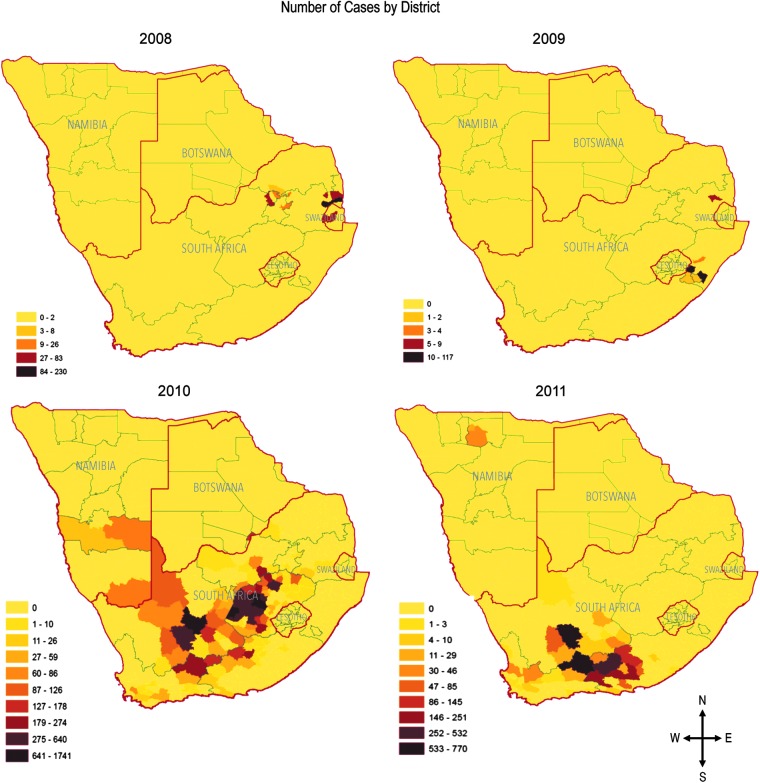
Number of Rift Valley fever (RVF) cases aggregated by subprovince for each epidemic year.

In 2008, outbreaks began in mid-January and continued through August, affecting the northeastern regions of South Africa and Swaziland. The 2009 outbreak lasted from February to May and was mostly concentrated in a small area in the south of Kwazulu-Natal Province. In 2010, the outbreak began in October, 2009, and continued through September, 2010, spanning most of South Africa, but was particularly concentrated in the interior regions, with some cases reported further west in Namibia and further north in Botswana. The 2011 epidemic began in December, 2010 and lasted through August, 2011; however, cases peaked in late April as compared to late February and mid-March as they had during the previous years. Most cases occurred in eastern and western Cape Provinces in the southwest of South Africa, but there was one outbreak reported in northern Namibia (Figs. 1 and 3).

### Environmental factors

**FIG. 4.**
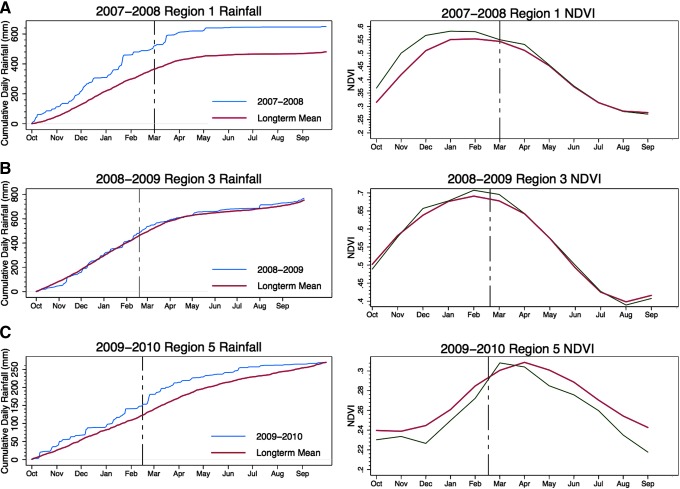
Long-term mean and daily cumulative rainfall (blue lines) and long-term mean and monthly cumulative normalized difference vegetation index (NDVI) (green lines) compared to first Rift Valley fever (RVF) case date (vertical black dotted line) for regions 1 (**A**), 3 (**B**), and 5 (**C**).

RVF followed periods of extended anomalously high rainfall (>60 days) in seven out of the nine predefined regions (Table 3). The average time between rainfall accumulation above average and the first case report was 115 days, ranging from 85 to 152 days, validating the findings from Kenya (Linthicum et al. 1985). However, in regions 3 and 6, rainfall was above average only for a short period of time before the first case appeared (Fig. 4). The amount of accumulated rainfall also varied widely between regions (Table 3).

**Table 3.** Environmental Factors and Case Numbers by Region

*Year*	*Region*	*Date*^a^	*RVF*^b^	*Days*^c^	*Rainfall amount*^d^	*NDVI above average*	*Outbreak locations*	*Cases*
2008	1	1-Oct-08	1-Mar-08	152	158.00	Yes	12	135
	2	1-Oct-08	14-Jan-08	105	82.43	Yes	15	328
2009	3	9-Feb-09	18-Feb-09	9	20.52	Yes	17	200
2010	4	1-Oct-09	19-Jan-10	110	57.77	Yes	265	9824
	5	11-Oct-09	15-Feb-10	127	27.60	No	137	2812
	8	2-Jan-10	10-May-10	128	12.52	No	9	37
2011	6	15-Dec-10	19-Jan-11	35	32.58	Yes	105	3888
	7	15-Dec-10	10-Mar-11	85	24.21	Yes	15	128
	9	28-Dec-10	4-Apr-11	97	317.32	Yes	2	73

^a^ Date daily cumulative mean rainfall exceeded average cumulative mean rainfall.

^b^ Date of first RVF case.

^c^ Days since daily cumulative mean rainfall exceeded average cumulative mean rainfall.

^d^ Cumulative rainfall amount above average since October 1^st^.

RVF, Rift Valley fever; NDVI, normalized difference vegetation index.

NDVI analyses revealed a pattern further from what was expected. Although seven of nine regions showed NDVI values above average prior to the first RVF case, they were not the same regions with above-average rainfall. Both regions 3 and 6, which had few days of above-average rainfall before the first RVF case was identified, had above-average NDVI at this time. Conversely, regions 5 and 8, which had anomalous rainfall at the time of the first RVF case, had below-average NDVI at that time (Fig. 4). This adds strength to the argument that other elements such as soil, topography, or manmade structures played a role in the 2008–2011 outbreaks. It also may suggest that there is a different NDVI threshold associated with an outbreak in southern Africa than eastern Africa.

**FIG. 5.**
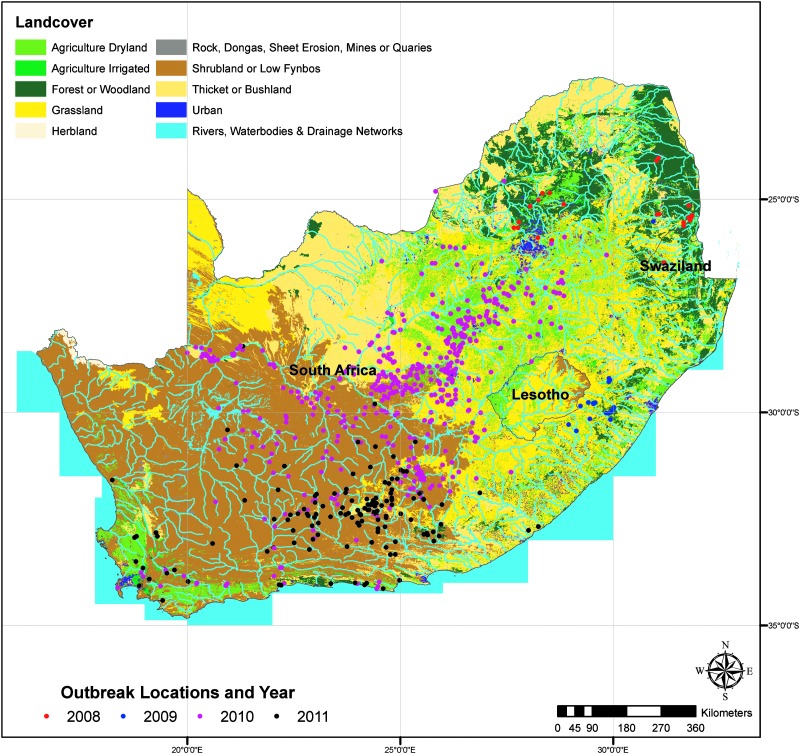
Reported Rift Valley fever (RVF) case locations in relation to Land Use/Land Cover.

Subsequent land use analyses revealed some differences between years; however, the majority of outbreaks occurred in shrubland, low fynbos, grassland, or agricultural areas. Even in regions where rainfall and NDVI were inconsistent, land use at case locations was similar to other regions. Overall, 37% of locations were areas of shrubland, low fynboes, or herbland—all from 2010 and 2011. Grassland regions were the next highest represented area, accounting for 20.6% of locations, followed by agriculture dryland (16.2%) and agriculture irrigated land (10.8%). The land use at outbreak locations in 2008 was notably different from the following years; more than half (55.2%) of the locations were categorized as forest, woodland, and forest plantation. This is compared to 2010 and 2011, where case locations were predominantly shrubland and grassland, accounting for 58.2% and 67.9% of locations, respectively (Table 4). Another feature that is evident is the proximity to water bodies, including rivers and drainage areas (Fig. 5). One example is the clustering of cases around the Orange River in 2010.

**Table 4.** Land Use at Unique Case Locations by Year for Outbreaks In South Africa^a^

	*2008*	*2009*	*2010*	*2011*	*Total*
Shruband, low fynbos, herbland			171 (34.1%)	82 (61.2%)	253 (37.0%)
Grassland	2 (6.9%)	9 (47.4%)	121 (24.1%)	9 (6.7%)	141 (20.6%)
Agriculture dryland	5 (17.2%)	2 (10.5%)	98 (19.5%)	6 (4.5%)	111 (16.2%)
Agriculture irrigated	5 (17.2%)	7 (36.8%)	44 (8.8%)	18 (13.4%)	74 (10.8%)
Thicket and bushland	1 (3.4%)	1 (5.3%)	41 (8.2%)	14 (10.4%)	57 (8.3%)
Other			27 (5.4%)	5 (3.7%)	32 (4.7%)
Forest, woodland, forest plantation	16 (55.2%)				16 (2.3%)
Total	29	19	502	134	684

^a^ Given as number of unique case locations *n*(%).

## Discussion

From 2008 to 2011, South Africa experienced the worst wave of RVF outbreaks in over 30 years. Throughout this 4-year period, every province in the country reported disease in livestock. In 2008 and 2009, the epidemics were smaller, associated with RVF lineage C, and the main species affected was cattle in the eastern exterior provinces, where there is a higher cattle density. The 2010 and 2011 epidemics were larger, associated with lineage H, and mainly affected sheep in the interior and southern provinces of South Africa (Grobbelaar et al. 2011). The case fatality rate was consistently higher among sheep every year with the exception of 2011.

The year 2011 marked the first time a large outbreak of RVF was detected and recorded in the Western Cape regions; disease had only occurred here once before during a small outbreak in 1983–1984 (Pienaar and Thompson 2013). Although 2011 was not as large as the outbreak in 2010, the disease did seem to be more severe, indicated by a high case fatality rate for all species. It is suspected that because it was the first appearance of disease in this region, immunity was lower due to a combination of first exposure, lack of vaccination, and more naïve and vulnerable breeds present in this region.

Although the potential for outbreaks existed, current methods using rainfall, NDVI, sea surface temperatures, and outgoing longwave radiation were unable to predict precisely areas that would be affected, especially in 2010 and 2011, indicating differences in environmental and RVF dynamics not only between eastern and southern Africa but also within southern Africa itself. We found that above-average rainfall for a period of 85–152 days preceded RVF outbreaks in seven out of nine regions over the 4 years. However, in two regions, RVF occurred less than 40 days after rainfall exceeded the long-term average. This is not indicative of RVF risk as previously seen in East Africa, where outbreaks have tended to occur after 50–60 days of anomalous rainfall (Anyamba et al. 2012). In addition to this, NDVI in regions 5 and 8 was below average, and there was some incongruence between NDVI and rainfall within the regions. This observation was unexpected considering the strong association between rainfall and NDVI in other parts of Africa and findings on RVF in East Africa (Linthicum et al. 1990, Nicholson et al. 1990, Tucker and Nicholson 1999, Anyamba et al. 2009).

One explanation for this is the large aggregated areas that were used to make rainfall and NDVI comparisons. Each of the nine regions was analyzed as if there was constant rainfall and NDVI. Although analyses comparing NDVI and rainfall at individual locations to the aggregated regions revealed similar trends, it has been reported that NDVI and rainfall correlations are highest at small scales because NDVI is also dependent on other factors, such as topography and soil type, that vary within our regions (Nicholson et al. 1990). It is plausible that water in shallow areas had accumulated, but was undetectable using large regions. Small geographic differences, such as being located at the bottom of an incline where water accumulates or near a water basin, could also increase the chance of mosquito breeding and RVF transmission. One place this is seen is along waterways in the Northern Cape province (Fig. 5). Future research aimed at characterizing these other factors, such as soil type, proximity to water, and topography, is needed.

Two other limitations were potentially missed data and no control group for further analyses. It is likely that some cases were missed, including asymptomatic infections. These animals would greatly add to the knowledge of risk factors but would not be captured. There also is a possibility of underreporting, especially in remote areas of Namibia and Botswana, where surveillance is weak. Two important factors not included were age of the animals that died and the abortion rate among herds of different species. Abortion and death in young animals are the most common outcomes of RVF. Adult fatality may be less correlated with RVF and its predictors than animal abortions would be and may be more indicative of breed or some other variable. If feasible, it would be helpful to collect more detailed information, including age of the animals, breed, and overall abortion rates. If we had his information on cases and controls, analyses could be conducted to identify risk factors for infection.

## Conclusion

In conclusion, above-normal rainfall and NDVI are still indicative of possible RVF disease activity; however, other ecological factors probably play a significant role in South Africa in relation to outbreak potential, such as geographic location relative to water bodies and surrounding land use. It will be important to identify these because vaccination is not recommended after the onset of an outbreak and is too expensive for many countries to maintain. An early warning system is currently the best method to prepare and take preventive action. If a warning is issued, countries have the power to prioritize and prepare, enabling proactive prevention like vaccination versus a reaction to fight an epidemic. As climate continues to change and shift toward more extremes, it is likely that vector populations will expand to new geographic locations, bringing with them many diseases that put human health and economic well-being at risk (Martin et al. 2008). Therefore, this work could have future applications, especially for diseases for which there is no effective vaccine.
